# From complexity to clarity: aging bone marrow niche in bone and blood regeneration and malignancy

**DOI:** 10.1038/s41413-026-00543-3

**Published:** 2026-05-18

**Authors:** Nainita Roy, Hanyu Liu, Allison L. Horenberg, Arvind P. Pathak, Junyu Chen, Saravana K. Ramasamy, Martine Cohen-Solal, Aline Bozec, Warren L. Grayson, Anjali P. Kusumbe

**Affiliations:** 1 Department of Pathology, New York University Grossman School of Medicine, New York, NY USA; 2 Tissue and Tumor Microenvironments Lab, Cancer Discovery and Regenerative Medicine Program, Lee Kong Chian School of Medicine, Nanyang Technological University, Singapore, Singapore; 3 Department of Biomedical Engineering, Johns Hopkins University School of Medicine, Baltimore, MD USA; 4 Translational Therapeutics & Regenerative Engineering Center, Johns Hopkins University School of Medicine, Baltimore, MD USA; 5 Institute for NanoBioTechnology, Johns Hopkins University, Baltimore, MD USA; 6 Russell H. Morgan Department of Radiology and Radiological Science, Johns Hopkins University School of Medicine, Baltimore, MD USA; 7 State Key Laboratory of Oral Diseases, National Center for Stomatology, National Clinical Research Center for Oral Diseases, Department of Prosthodontics, West China Hospital of Stomatology, Sichuan University, Chengdu, China; 8 Lee Kong Chian School of Medicine, Nanyang Technological University, Singapore, Singapore; 9 Multidisciplinary Institute of Ageing (MIA-Portugal), University of Coimbra, Coimbra, Portugal; 10 Department of Rheumatology and Reference Center for Rare Bone Diseases, Hospital Lariboisière, Paris, France; 11 Inserm U1132 Bioscar, Université Paris Cité, Centre Viggo Petersen Hôpital Lariboisière, APHP.Nord, Paris, France; 12 Department of Internal Medicine 3, Friedrich-Alexander-University Erlangen-Nürnberg (FAU) and Universitätsklinikum Erlangen, Erlangen, Germany; 13 Deutsches Zentrum Immuntherapie (DZI), Friedrich-Alexander-University Erlangen-Nürnberg (FAU) and Universitätsklinikum Erlangen, Erlangen, Germany; 14 Department of Materials Science and Engineering, Johns Hopkins University, Baltimore, MD USA; 15 Department of Chemical & Biomolecular Engineering, Johns Hopkins University, Baltimore, MD USA

**Keywords:** Cancer, Bone cancer

## Abstract

The bone marrow niche (BMN) plays a central role in regulating hematopoietic stem-cell (HSC) maintenance, lineage commitment, and immune homeostasis, while also supporting osteogenesis and maintaining skeletal integrity. Once considered static, the BMN is now recognized as a dynamic and responsive microenvironment that integrates local signals and systemic cues to meet physiological demands and respond to stress. Aging causes profound and progressive changes to this niche, leading to functional decline across both hematopoietic and stromal compartments. Recent advances in high-resolution imaging, single-cell and spatial transcriptomics, and in vivo lineage tracing have revealed remarkable heterogeneity and plasticity within the vascular and mesenchymal elements of this niche. Yet, key questions remain unresolved, including the identity and hierarchy of mesenchymal and osteolineage cells, the specialization of subsets of endothelial cells, the integration of systemic regulation, and whether the aging bone marrow acts as a driver or a passenger in malignancy and chronic inflammation. This review revisits current models of the BMN, with a focus on the reciprocal interactions between osteogenic cells and specialized vasculature, and how their disruption during aging impairs hematopoietic output and skeletal remodeling. We also examine how systemic factors such as neural input, metabolic status, and inflammatory signaling influence the aging of the BMN. Finally, we highlight emerging translational platforms, including iPSC-derived bone marrow organoids, engineered niches/hydrogels, and vascularized organ-on-chip systems, that enable mechanistic testing of rejuvenation strategies. Together, these insights have the potential to pave the way toward targeted interventions that restore the function of the BMN and promote healthy aging of the bone and blood systems.

## Introduction

The BMN within the skeletal system is a highly dynamic and functionally specialized tissue that governs hematopoietic stem-cell (HSC) fate.^1^ It not only maintains hematopoietic homeostasis but also coordinates regenerative responses and immune regulation. Far from being a passive scaffold embedded in bone, the BMN actively integrates systemic and local signals to fine-tune hematopoiesis across physiological states.^2^

Emerging evidence demonstrates how the BMN is profoundly influenced by aging and pathological remodeling. Age-related niche dysfunction and niche-driven contributions to hematological malignancies are increasingly recognized,^3^ yet the underlying mechanisms remain incompletely understood.^4^ While recent advances in single-cell transcriptomics,^5^ spatial profiling,^6,7^ and in vivo lineage tracing^8,9^ have revealed new layers of complexity in niche organization, there remain key unanswered questions regarding cell identity, lineage hierarchy, and niche function.^10,11^

Despite major technological progress, the field has been constrained by some key unresolved issues.^12^ These stem not only from the biological heterogeneity of bone marrow stromal and vascular compartments, but also from divergent experimental models and analytical frameworks. This has had significant translational consequences: interventions to extend lifespan or treat malignancies that target bone-derived stromal elements require a unified framework that the field currently lacks.

This review aims to critically examine some of these outstanding questions. Rather than offering definitive solutions, we highlight the principal areas where perspectives diverge and explore their underlying causes, whether methodological, conceptual, or contextual. By doing so, we hope to highlight opportunities to bridge current gaps in our knowledge and foster a more cohesive understanding of BMN biology.

## Mesenchymal identity and hierarchy in the BMN: unresolved identities

**Fig. 1:**
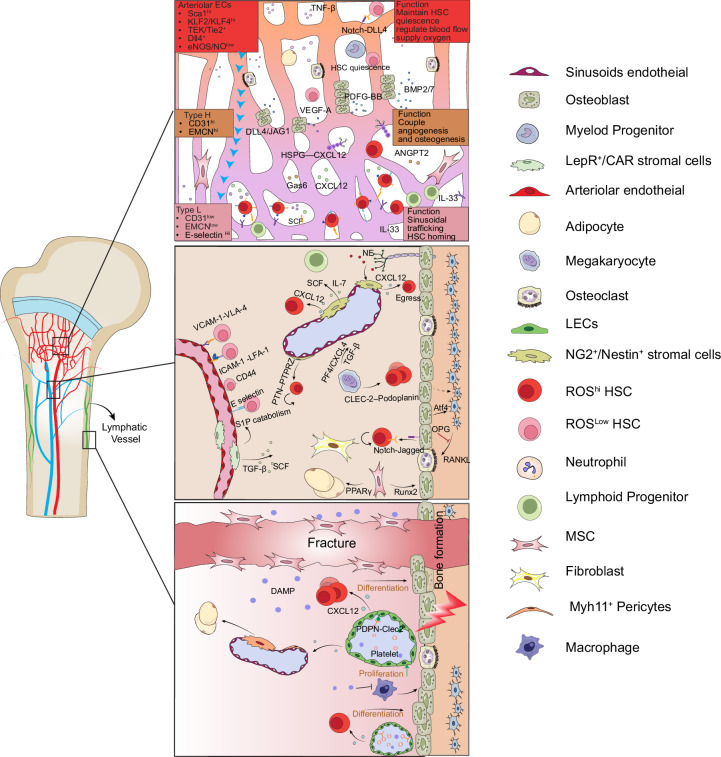
Schematic illustration of the architecture of the bone marrow niche. Endothelial cells within the bone marrow niche form highly heterogeneous populations that are based on genotype, phenotype, and functions. Vasculature includes arterioles, H-type, and sinusoidal (L-type) vessels. Arteriole vessels maintain blood flow and preserve HSC quiescence; H-type vessels couple angiogenesis and osteogenesis at the metaphysis region of the bone. Sinusoidal vessels facilitate immune cell trafficking and HSC homing in the bone marrow niche. Other key niche components, including mesenchymal stromal cells, osteoblasts, and hematopoietic stem cells, adipocytes, and lymphoid progenitors, interact with endothelial cells through adhesion molecules and paracrine or juxtacrine signaling pathways(SCF, CXCL12, notch, PDGF-BB, IL-33) to maintain niche homeostasis and regulate stem-cell maintenance

Mesenchymal stromal cells (MSCs) provide the structural and molecular foundation for the hematopoietic stem-cell (HSC) niche.^1,13^ They form an extracellular scaffold and provide regulatory cues in the form of cell–cell interactions, cytokines, chemokines, and extracellular matrix (ECM) components to support HSC maintenance, differentiation, and regeneration.^10^ Decades of research on mesenchymal populations in the bone marrow (BM) have revealed important advances but also left open questions regarding the identity, lineage relationships, and functional hierarchy of MSCs. The availability of single-cell RNAseq (scRNA-seq), spatial transcriptomics, and lineage-tracing data has accelerated progress but also highlighted heterogeneity that complicates classical definitions of cell identity.^6,14^ A central issue in bone biology is the identity and organization of mesenchymal populations that constitute the hematopoietic niche. Although originally conceptualized as osteoblast-driven,^15^ the niche is now recognized to be primarily perivascular,^1,16^ composed of stromal cells that regulate HSC maintenance and differentiation. Most HSCs are situated in proximity to sinusoidal capillaries or arterioles,^17^ where endothelial and adjacent stromal cells provide both survival and retention signals. Within these regions, CXCL12-abundant reticular (CAR) cells, Leptin receptor-positive (LepR^+^) stromal cells, and Nestin^+^ MSC-like cells secrete factors essential for HSC support.^10,16,18^ LepR^+^ stromal cells, in particular, are widely regarded as key producers of CXCL12 and stem-cell factor (SCF), essential for HSC quiescence and retention.^19^ Yet single-cell and spatial transcriptomic studies have revealed that the LepR^+^ compartment is heterogeneous, encompassing subtypes with divergent roles in lymphoid, erythroid, and myeloid support, as well as immune and vascular modulation.^6,20^ This diversity obfuscates the classical notion of a linear stromal hierarchy and complicates the identification of a single dominant niche population. See Fig. 1 for an overview of bone-vascular architecture and its stromal partners, highlighting arteriolar–sinusoidal gradients, perivascular stromal subsets (LepR^+^, CAR, Nestin^+^), and their principal HSC-regulatory cues. This figure emphasizes how endothelial and mesenchymal heterogeneity underlie context-dependent support of HSC quiescence, retention, and activation.

Moreover, functional definitions are often context-specific: subsets that support HSCs during homeostasis may behave differently under stress, inflammation, or aging.^21,22^ In aged BM, for example, LepR^+^ MSCs become biased toward adipogenesis^4^ and pro-inflammatory, resulting in impaired HSC support and hematopoietic biases.^9,14,23,24^ These dynamic transitions, together with the lack of consensus on reliable surface markers and lineage-tracing tools, have limited the field’s ability to establish a unified mesenchymal hierarchy. In summary, the mesenchymal compartment of the BMN comprises a heterogeneous and dynamic population of cells whose identity, lineage relationships, and functions vary with physiological context. Moving forward, integrating transcriptional, spatial, and lineage-tracing datasets along with developing a standardized nomenclature will be essential to resolve current uncertainties and establish a coherent framework for mesenchymal biology in the bone marrow.

The plasticity of MSCs that is essential for normal niche function also makes them susceptible to malignant reprogramming.^25^ In hematologic cancers, and multiple myeloma (MM) in particular, MSCs are reprogrammed toward tumor-supportive phenotypes that bolster malignant outgrowth, remodel the microenvironment, and promote therapeutic resistance. Patient-derived MSCs display defective osteogenesis and increased production of IL-6, CXCL12, VEGF, IGF-1, Activin A, and others, which in turn promote plasma cell proliferation, survival, and drug resistance.^26^ MM cell-derived exosomes can also reprogram MSCs, for example, by delivering miR-21 and miR-146a to drive IL-6/CCL2 secretion and promote malignant cell growth and survival.^27–29^ In addition to cytokines and exosomes, MSC–tumor crosstalk also involves Notch and adhesion pathways (CXCL12–CXCR4, VLA-4/VCAM-1), which tether plasma cells to perivascular niches and activate PI3K–Akt/NF-κB signaling, which further amplifies these survival and drug-resistance programs.^30–33^ Reciprocal paracrine signaling also promotes osteoclastogenesis (via RANKL/M-CSF) and inhibits osteoblasts, leading to the osteolytic bone disease that is characteristic of MM.^34,35^ Finally, MSC-produced cytokines and immunomodulators (TGF-β, PGE2, IDO, HLA-G) remodel the vascular and immune compartments to form an immune-privileged niche that directly suppresses T cell and NK cell antitumor activity.^36,37^ The combined effects of these pathways are a protective and growth-promoting MM microenvironment that contributes to minimal residual disease (MRD) and relapse. Thus, MSC–tumor crosstalk (including but not limited to CXCR4, VLA-4, RANKL, and exosome pathways) is an increasingly recognized therapeutic target in MM.^38–40^

## Vascular niches in bone and underlying complexities

The vasculature does not merely support the bone marrow niche; it also plays a role in defining HSC fate through spatially patterned angiocrine signals that rival stromal contributions in complexity and consequence. In addition to stromal and osteolineage cells, the vasculature is an integral and dynamic component of the HSC niche.^41^ Most of the studies that describe the vascular niche have been using long bones. Classically, BM endothelial cells (BMECs) have been broadly divided into arteriolar and sinusoidal subtypes based on presumed functional dichotomy, quiescence-supporting, low-permeability, arteriolar endothelial cells (AECs) localized to endosteal regions versus highly permeable, fenestrated sinusoidal endothelial cells (SECs) that support HSC activation, trafficking, and immune surveillance.^17,42^ This dichotomy provided an early framework for vascular regulation of hematopoiesis. However, recent work suggests that such compartmentalization oversimplifies the complexity, plasticity, and integration of BMECs within the niche.^41^ The traditional view of AECs as a low ROS, CXCL12- and SCF-rich quiescent niche versus SECs as immunologically and metabolically permissive hubs supporting HSC differentiation and cell migration has become more nuanced.^9,10,42^ Single-cell transcriptomics, spatial imaging, and genetic fate mapping data indicate that endothelial identity is neither fixed nor strictly binary, particularly under conditions of stress, inflammation, aging, and regeneration.^5,9,14^ In the past decade, scRNA-seq and spatial multiomics data have revealed previously unappreciated BMEC heterogeneity with Type-H, Type-L, transitional, periarteriolar, and perisinusoidal endothelial subtypes with overlapping molecular identity and incompletely defined functions.^9,43,44^ The Type-H endothelial cell population, which is defined by high expression of CD31 and Endomucin and found in regions enriched with osteoprogenitor cells at metaphyseal and endosteal niches, couples angiogenesis and osteogenesis to support hematopoietic reconstitution after injury.^9,43,45^ Type-H vessels are prominent in young and regenerating marrow but decline sharply with aging,^45–48^ with work by Zhang et al.,^49^ showing that the basement membrane is remodeled with increased ECM stiffness and a subsequent loss of spatial coupling between osteoblast precursors and ECs. This physical disruption of niche organization and compartmentalization contributes to a loss of angiocrine support for HSCs, with corresponding deficits in function.^50^ Aged BM vasculature exhibits reduced SCF/CXCL12 production and increased leakiness, features linked to HSC functional decline and myeloid skewing.^51,52^

In parallel, SECs are now recognized to be far more active than previously appreciated. Transcriptomic profiling has revealed that SECs display context-dependent heterogeneity, including stress-responsive states during hematopoietic regeneration and inflammation.^5,14,53^ Under inflammatory challenge, bone marrow endothelial cells upregulate cytokines such as IL-6 and G-CSF, while downregulating retention factors including CXCL12 and adhesion molecules like VCAM-1, thereby influencing HSC mobilization, proliferation, and immune cell crosstalk.^54,55^ Aging and leukemia both induce sinusoidal dilation, leakiness, and interferon/inflammatory remodeling, impairing HSC quiescence and biasing hematopoiesis.^45,56^

Moreover, the idea of a privileged, static arteriolar niche as the singular source of HSC quiescence has been revised. In fact, bone marrow arterioles remodel dynamically under stress, irradiation, and aging, and are associated with altered vascular permeability, integrity, and angiocrine signaling.^9,43,45,47^ Ramasamy et al. showed that endothelial cells coordinate angiogenesis with osteogenesis, and periarteriolar endothelial cells directly support osteoprogenitors via Notch and VEGF-dependent angiocrine mechanisms.^43,47^ While Wnt signaling is a well-established master regulator of osteoblast differentiation and bone homeostasis,^57,58^ Wnt’s function within BMEC subsets has been largely deduced from studies of other vascular beds where endothelial Wnt/β-catenin activity maintains vascular integrity,^59^ though its marrow-specific role remains incompletely defined.

In addition to hierarchically regulating HSCs, endothelial cells were recently shown to play a role in immune tolerance. A recent study identified specialized sinusoidal and capillary regions co-expressing PD-L1, CD200, and MHC molecules that provided immune protection to primitive HSCs.^60^ These immune-privileged, nitric oxide-high (NO^hi^) HSCs resided in a distinct subset of ciliated, CD200^hi^ capillaries at the metaphysis and maintained long-term reconstitution. Disruption of CD200 or ciliary protein IFT20 in these endothelial cells impaired eNOS signaling and led to decreased autophagy in NO^hi^ HSCs and a loss of their long-term reconstitution potential. Importantly, these findings challenge the assumption that Type-H and sinusoidal vessels serve as the default niche for long-term HSCs, and instead identify a novel vascular compartment marked by immune protection and hierarchical control.^60^

Another emerging dimension of vascular biology in bone is lymphatics. Recent studies using high-resolution imaging and lineage tracing have overturned the dogma that bone lacks lymphatics. Lymphatic endothelial cells (LECs) have now been identified in bones, where they expand after injury and actively interact with stromal, vascular, and hematopoietic cells.^61^ Direct genetic evidence in mouse models and light-sheet fluorescence microscopy has identified lymphatic vessels in the cortical bone and marrow compartments.^61^ Three-dimensional PET imaging also hints at the presence of lymphatics in human skull bone marrow.^62^ Single-cell sequencing experiments have confirmed the existence of LECs in bone.^63^ IL-6-mediated lymphangiogenesis induced by injury activates bone-resident LECs to produce lymphangiocrine signals such as CXCL12 that modulate progenitor activation, perivascular recruitment, and hematopoietic regeneration.^61^ Spatial transcriptomics studies in response to bone fracture and osteotomy have also uncovered Prox1^+^Pecam1^+^Ptprc^-^LECs specifically localized in the cortical bone, marrow compartment, and newly formed regenerating woven bone.^64^ Additionally, tissue engineering strategies have incorporated the recruitment of LECs in 3D-printed scaffolds to show that the orchestration of lymphangiogenesis, angiogenesis, and osteogenesis through LEC-mediated HIF-1α signaling promotes rapid bone regeneration.^65^ Likewise, dual-electroactive microspheres potentiate vascularization, lymphangiogenesis, and neurogenesis to similarly promote bone repair.^7^ Together, these studies identify the essential roles of LECs in preventing medication-related osteonecrosis of the jaw,^66,67^ enabling hip implant healing, allowing alveolar bone regeneration in periodontitis,^68^ and promoting bone regeneration in aged skeletons.^69,70^ In the aging skeleton, the functional decline of bone marrow lymphatics is a key process that mirrors and likely contributes to hematopoietic dysfunction. LECs experience age-associated genotoxic stress that impairs lymphangiocrine signaling, resulting in decreased lymphangiogenesis and defective bone regeneration in the elderly.^61^ Importantly, young LEC transplantation restores lymphatic expansion and significantly enhances both bone formation and hematopoietic regeneration in old mice, proposing the rejuvenation of LECs as a potential treatment approach.^61,71^ Intraosseous lymphatics may act as early responders to systemic insults, modulating inflammaging and potentially providing routes for tumor dissemination or leukemic remodeling.^66^ Studies on bisphosphonate-related osteonecrosis reveal pronounced rarefaction of both type-H blood vessels and lymphatic vessels in affected mandibular bone.^72^ Furthermore, lymphatic drainage plays a critical role in fracture repair: lymphatic platelet thrombosis can obstruct lymph flow, causing insufficient drainage and impaired healing, while enhancing lymphatic drainage reduces neutrophils, increases reparative M2-like macrophages, and supports osteoblast survival.^71^ Open questions include their developmental origins, anatomical heterogeneity, and integration with specialized vascular subsets. Methodologically, incorporating lymphatic endpoints into aging and leukemia models will be crucial to defining causality and therapeutic windows. In sum, the vascular niche is not a binary arteriolar–sinusoidal system but a stratified, transcriptionally diverse, and dynamically reconfigurable network. Endothelial identity is shaped by developmental stage, anatomical context, and physiological perturbation, and endothelial cells are not merely a passive scaffold but actively contribute to niche remodeling, immune crosstalk, and HSC hierarchy. Aging, inflammation, and disease disrupt this balance, leading to altered niche signals, niche physical disorganization, and hematopoietic decline. Despite progress, consensus is still lacking on EC subtype definitions, marker specificity, and functional spatial coupling. Moving forward, functional assays, such as selective ablation/induction of EC subpopulations^73^ combined with single-cell multiomics and high-resolution spatial imaging, will be essential to build an integrated model of vascular niche biology and enable rational therapeutic targeting in regeneration, transplantation, and cancer.

Importantly, vascular remodeling is not just structural but immunologic: enhanced permeability and altered angiocrine output can re-pattern immune cell trafficking and cytokine exposure within the marrow. The described vascular and lymphatic networks form the structural and molecular basis for niche function. Niche compartments are not, however, subjected to aging in isolation. As will be highlighted in the next section, aging leads to profound remodeling of the BMN by highly interconnected processes, including vascular rarefaction, chronic inflammation, sympathetic denervation, and stromal dysfunction. Dissecting the temporal sequence and causal relationships between these aging hallmarks represents a major challenge in the field.

## The aging and malignant BMN: instigator or reactor?

**Fig. 2:**
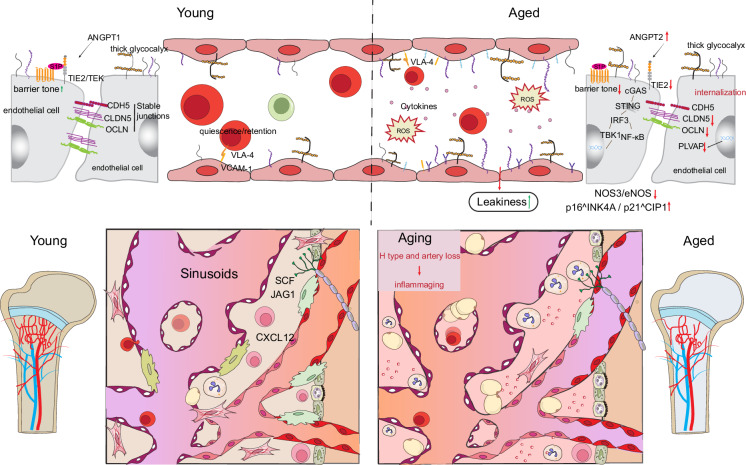
Aging remodels the bone marrow niche. Compared with the young bone marrow niche, the aged niche demonstrated leakage of bone marrow vasculature, elevated pro-inflammatory signaling, increased ROS, cellular senescence, adipocyte accumulation, MSC dysfunction, HSC exhaustion, and immune response. Collectively, those contribute to damage to the bone marrow microenvironment

As the skeleton ages, the BMN undergoes significant remodeling, accompanied by progressive hematopoietic decline, manifested as increased myeloid bias, reduced lymphopoiesis, impaired regenerative responses, and heightened susceptibility to hematologic malignancies.^12,45,74,75^ Fig. 2 provides a one-glance overview of aging-associated niche remodeling across vasculature, stroma, innervation, and immune tone. But a central question persists: does the aging niche deteriorate because HSCs fail, or do HSCs fail because the niche deteriorates first? The answer increasingly appears to be both.

### Inflammaging and cytokine signaling

A defining feature of the aging bone marrow is low-grade chronic inflammation, or “inflammaging.” Aged niches exhibit elevated levels of inflammatory cytokines, including IL-1β, TNF-α, IL-6, and type I interferons, which drive HSCs toward myeloid differentiation and impair self-renewal.^48,76–84^ While the source of these inflammatory cues remains debated, ranging from senescent stromal cells to systemic inflammatory burden, their impact on hematopoiesis is clear. Chronic IL-1 signaling has been implicated in maintaining a state of persistent emergency myelopoiesis^13^ and exhausting the HSC pool. Strikingly, pharmacological blockade of IL-1 signaling in aged mice has been shown to reduce marrow inflammation and restore aspects of hematopoietic function.^78^ These findings highlight the plasticity of the aging niche and underscore inflammation as a tractable therapeutic target^78^ (see Fig. 2). As inflammaging is built from repeated cytokine modules from various niche and immune sources, we provide an overview of important inflammatory mediators, their probable cellular sources, evidence from aging models and functional implications for the HSC and stromal/endothelial compartments in Table 1. Inflammaging is not merely a correlate of bone marrow decline, but rather an active driver that can be pharmacologically interrupted, revealing the aging niche as a therapeutically modifiable target rather than an irreversible endpoint.

**Table 1:** Key niche-related signals involved in age-related dysfunction of the bone marrow

Key cytokine/signal	Primary cellular source(s)	The change of signals	Effects on HSCs	Effects on Niche Cells	Evidence from aging models
CCL2 (MCP-1)	Senescent MSCs, stromal SASP programs	↑ regulated	Indirect: recruits inflammatory monocytes → chronic HSC inflammatory exposure	Promotes macrophage accumulation, osteoclastogenesis, stromal senescence feedback	Human ex vivo^219,220^
CCL5 (RANTES)	Immune cells, stromal cells	↑ regulated	Associated with aged inflammatory HSC states; CCR5 marks an HSC subset and relates to CCL5-rich environments	immune recruitment/activation	Physiologic aging mouse model; chemokine/HSC subset studies^221^
CXCL12 (SDF-1)	LepR^+^/CAR stromal cells and endothelial compartments; lymphatic ECs during stress repair	↓ expression and spatial disorganization	Reduced HSC retention, altered quiescence, impaired homing	CXCL12-dependent perivascular support programs; stromal differentiation effects are context-specific	Physiologic aging mouse model^61^
HIF-1 α	Endothelial cells, HSC	↓ regulated	Loss of type-H vascular niche support with age contributes to impaired osteo-angiogenic coupling and HSC-support factor decline	H-type vessel loss. Angiogensis reduce	Physiologic aging mouse model^95^
IL-1β/IL-1α	Myeloid cells, Senescent MSCs, aged macrophages, stressed endothelial cells	↑ regulated	Loss of quiescence, myeloid bias, emergency myelopoiesis, exhaustion of self-renewal capacity	MSC senescence, osteogenic suppression, vascular inflammation, SASP amplification	Physiologic aging mouse model; IL-1 blockade in aged mice ameliorates niche deterioration and restores blood production^12^
IL-6	CXCL12-abundant reticular cells, Senescent MSCs, adipocytes, endothelial cells, osteoblasts	↑ regulated	HSC cycling, megakaryocyte/platelet bias, emergency granulopoiesis, STAT3 activation	Stromal activation, osteoclastogenesis, pro-inflammatory phenotype, gp130 signaling	Chronic low-grade IL-6 in aged mice correlates with megakaryocyte expansion and loss of lymphopoiesis^105^
NLRP3 inflammasome	Immune cells; aged HSCs	↑ regulated	Decline in regenerative capacity; inhibition of HSCs maintenance/ regeneration	Niche impact via IL-1β/IL-18 production and inflammatory amplification (context dependent)	Physiologic (natural) aging^222^
Notch ligands (DLL4, JAG1)	Type-H endothelial cells (metaphysis/endosteal), arterial ECs	↓ signaling activity	Reduced supportive vascular niches; impaired HSC maintenance	Loss of type-H vessels; reduced SCF production; impaired osteo-angiogenic coupling	Naturally aged mice^95^
Angiocrine factors (ANGPT1, RSPO3)	Endothelial cells	↓ protective angiocrine output	Loss of HSC quiescence, increased cycling	Shift from regenerative to inflammatory angiocrine profile	Physiologic aging mouse model^73,95^
Wnt signaling (canonical & non-canonical)	Osteoblasts, stromal cells	Dysregulated (often ↑ but ineffective)	HSC exhaustion, impaired differentiation balance	Excess or mislocalized Wnt signaling is deleterious	Physiologic aging mouse model^223,224^
ROS / oxidative stress signals	Endothelial cells, stromal niche	↑ oxidative burden	DNA damage, loss of HSC self-renewal	Linked to vascular dysfunction and reduced perfusion	Physiologic aging mouse model; mitochondrial oxidative stress–driven bone and niche aging mouse model^225,226^
Adhesion molecules (VCAM-1, integrins)	Endothelial and stromal cells	Altered expression and distribution	Impaired HSC anchorage and polarity	Affects niche localization and asymmetric division	ICAM-1-deficient mice Physiologic aging mouse model^79^
PDGF-BB	Pericytes, endothelial-associated perivascular cells; also pre-osteoclast lineage cells in bone coupling niches	↓ regulated	Indirect support of HSC maintenance via vascular niche integrity; decline contributes to reduced HSC-supportive microenvironments	aging-associated reduction leads to loss of type-H vessels and impaired bone-vascular niche function	Physiologic aging mouse model^95^
S100A8/S100A9 (alarmins; DAMPs)	Inflammatory myeloid cells; Stressed stromal compartments	↑ regulated	Induce genotoxic/inflammatory stress programs in HSPCs; dysfunctional haematopoiesis	Promote inflammatory stromal remodeling	Naturally aged mouse and Human donor aged BM samples Mouse inflammatory stress and Mouse clonal hematopoiesis; Mouse niche senescence models, Chronic inflammatory disease patients^227^
SCF (KITL)	Endothelial cells, LepR⁺ stromal cells	↓ availability (especially membrane-bound SCF)	Reduced HSC maintenance and survival signaling	Reflects deterioration of vascular niche health	Physiologic aging mouse model^95^
Thrombospondin-1	Megakaryocytes, aged platelets, endothelial cells, osteoblasts	↑ regulated	Inflammatory priming, enhanced ROS, myeloid bias, reduced reconstitution	TGF-β activation, ECM remodeling, stromal senescence, fibrosis	Thbs1 knockout mice exhibit preserved HSC activity and balanced lineage potential during aging^228^
TNF-α	Macrophages, Neutrophils, T cells, senescent stromal cells	↑ regulated	Impaired self-renewal, myeloid skewing, ROS accumulation, HSC survival, context-dependent activation	Endothelial dysfunction, MSC adipogenesis, osteoclast activation, NF-κB signaling	TNF-α drives clonal expansion of *Tet2*-deficient HSCs^201^
Type I IFNs					
(IFN-α/β)	Senescent cells (cGAS-STING), endothelial cells, immune cells	↑ regulated	Chronic activation, proliferative exhaustion, ISG signature, clonal selection	Endothelial activation, vascular leakiness, SASP amplification, DNA damage response	Chronic stress models (poly(I:C)) in mice show that repeated IFN exposure permanently depletes the LT-HSC pool^229^
VEGFA	Endothelial cells, osteoblast-lineage cells, stromal cells in the endosteal/vascular niche	↓ regulated	Indirect effect: reduced vascular support leads to impaired HSC maintenance and niche function	Aging-associated loss of type-H vessels, reduced angiogenesis, and impaired osteo-angiogenic coupling	Physiologic aging mouse model^95^
VEGFC	Lymphatic endothelial cells (LECs) in bone marrow	↓ regulated	Reduced CXCL12-mediated HSC support during aging or genotoxic stress; impaired hematopoietic regeneration	Decline of LEC populations and lymphatic niche function; reduced CXCL12 production and vascular repair capacity	Physiologic aging mouse model^61^
Sympathetic nervous system signaling (β-adrenergic)	Nerve fibers acting on stromal cells	Dysregulated circadian signaling affects	Reduced rhythmic mobilization; impaired niche synchronization; contributes to functional decline and altered hematopoietic output with aging	Niche denervation leads to reduced CXCL12 rhythmic expression, stromal dysfunction, increased inflammatory tone, and impaired vascular–stromal coordination	Physiologic aging mouse model^86^

Therapeutic Implications: Targeting inflammatory pathways represents a promising strategy for BMN rejuvenation. IL-1 receptor antagonists (anakinra), TNF inhibitors (etanercept), and senolytics (dasatinib + quercetin) have shown efficacy in preclinical models and are being evaluated for hematopoietic restoration. See Table 2 for comprehensive therapeutic strategies

### Neuronal decline

Sympathetic innervation, crucial for circadian HSC trafficking, vascular tone, and osteogenesis, has emerged as another critical, yet underexplored dimension of age-related remodeling.^85^ Experimental denervation in young mice reproduces several hallmarks of aged marrow, including vascular disintegration, increased marrow adiposity, and HSC activation.^86^ Conversely, stimulation of β3-adrenergic receptors in aged mice restores aspects of sympathetic signaling, improving both niche integrity and HSC function. These findings suggest that neuronal attrition is an upstream event in niche aging, though its relationship to vascular and inflammatory changes remains unresolved.

### Systemic regulation

**Fig. 3:**
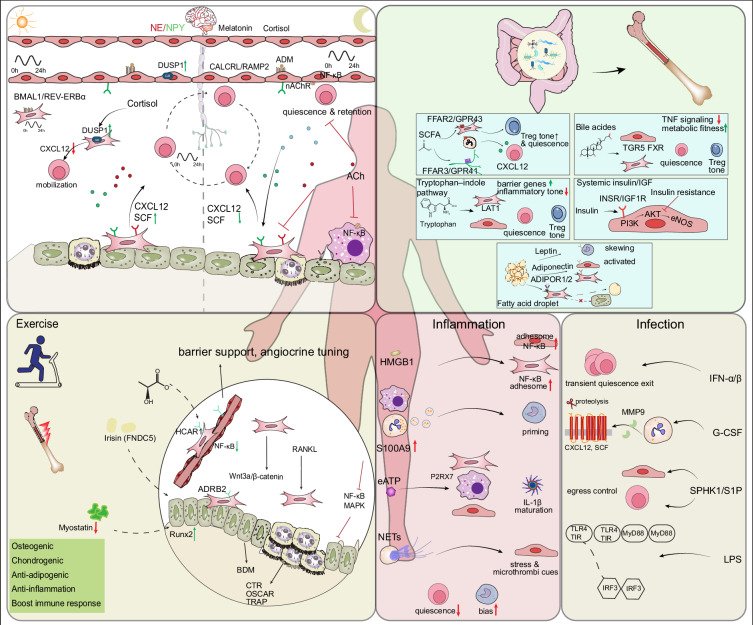
Systemic regulation of the bone marrow niche. The bone marrow niche is dynamically regulated by circadian rhythms, metabolic cues from the gut, physical activity, and inflammatory signals. Circadian genes (BMAL1) regulated cellular rhythmicity and sympathetic neurotransmitters, which directly target MSCs, endothelial cells, and HSCs, coordinate HSC quiescence and mobilization. ADM and acetylcholine promote HSC retention, while NPY and NE signaling enhance egress. Gut-derived microbial and metabolic ligands also modulate niche homeostasis, short-chain fatty acids (via FFAR2/FFAR3) support Treg tone and HSC quiescence, whereas high-fat diet and LEPR signaling drive inflammatory myeloid bias and derived MSCs differentiate into adipocytes. Gut-Microbiota-induced tryptophan metabolites and bile acids activate their receptors on the EC and MSC to promote metabolic fitness and reduce inflammatory factors. Exercise remodels the niche 2-adrenergic signaling, and exercise-induced irisin and lactate promote long-term niche fitness. Sterile inflammation reshapes the niche via DAMPs, cytokines, and NETs, resulting in reduced HSC quiescence and skewing differentiation toward myeloid lineages. During infection, type I interferon, G-CSF, and S1P gradients activate and mobilize HSCs, causing exhaustion and intercellular antiviral reprogramming

Systemic factors also profoundly affect the aging niche with direct effects of circulating cytokines, metabolic, and hormonal signals on stromal and vascular cells capable of promoting or partially counteracting aging.^45^ Figure 3 summarizes endocrine, metabolic, neural, inflammatory, and microbiome inputs converging on endothelial and stromal circuits (e.g., SCF–c-Kit, Jagged–Notch, CXCL12), outlining upstream (system-level) and downstream (niche-intrinsic) intervention points. The heterochronic parabiosis model, for instance, demonstrates that a young systemic environment can at least partially restore aged HSC function, though intrinsic deficits in old HSCs remain.^87–89^ Dietary and microbiome-derived metabolites have been shown to intersect with niche biology as well. Short-chain fatty acids (SCFAs) have the ability to modulate hematopoiesis through GPR41/43 signaling^90^ and microbial-derived lactate has been shown to increase SCF expression in LepR^+^ stromal cells through GPR81 signaling^91^ (see Fig. 3). Consistent with this, high-fat-diet–driven remodeling of the gut microbiota has been demonstrated to alter the bone marrow niche and skew HSC differentiation, with effects partially reversible by antibiotics or transferable by fecal transplant.^92^ These examples illustrate how systemic cues intersect with local niche biology, though they also complicate efforts to establish causality.

### Vascular remodeling and rarefaction

Structural vascular decline is another prominent hallmark of the aging niche.^45,47^ Arteriolar integrity wanes, sinusoidal leakiness increases, and endothelial production of CXCL12 and SCF diminishes.^43,47,73^ Type-H endothelium, which couples angiogenesis and osteogenesis, is lost with age, leading to decoupling of osteoprogenitors and endothelial cells.^43,45,47,52^

Functionally, impaired vascular support, along with inflammatory remodeling and sympathetic denervation, reduces CXCL12/SCF availability and HSC quiescence and leads to myeloid skewed differentiation of stromal cells.^12,53,93,94^ However, the extent and directionality of this remodeling remain an open question. On the one hand, rarefaction and leakiness of sinusoidal endothelium have been detected before any signs of hematopoietic collapse in aging^45,95^ and therefore may represent an upstream cause of hematopoietic decline that is driven by vascular dysfunction. On the other hand, studies of clonal hematopoiesis indicate that mutant HSCs themselves stress the niche environment to drive and exaggerate age-associated remodeling.^96,97^ Importantly, some studies also indicate that specific stromal or vascular compartments retain their capacity to support hematopoiesis or even adapt in compensatory ways. For example, periarteriolar NG2^+^ pericytes, known to maintain HSC quiescence, are relatively preserved during aging and may compensate for declining sinusoidal support.^42^ Similarly, recent work suggests that subsets of endothelial cells upregulate angiocrine factors in response to inflammatory cues, helping to buffer the aged niche against complete collapse.^73^ Functionally, old HSCs acquire a more “youthful” transcriptomic state upon transplantation into a young host, but they maintain the self-renewal defect and myeloid bias when tested in functional assays such as competitive repopulation and long-term engraftment.^98,99^ This suggests that the changes in gene expression of aged HSCs are environmentally modulated, while their functional defects are primarily cell-intrinsic and stable.^100^ In this respect, transplanting young HSCs into aged marrow often results in only partial and transient improvement, highlighting the lasting impact of the aged microenvironment even in the context of a supportive host.^98^ Together, these observations support a model in which HSCs and niches age in concert, mutually reinforcing decline.

Lineage-tracing approaches have introduced their own layer of excitement in the field, along with complexity. BM stromal–restricted Cre drivers (e.g., Osx-Cre targeting osteoprogenitors and Lepr-Cre targeting perisinusoidal stromal cells) have implicated critical roles for both these compartments in supporting the niche.^74^ However, interpretation of these studies has also been complicated by the fact that Osx-Cre and Lepr-Cre lines can have an unwanted lack of specificity or recombine in non-target populations (including in postnatal or non-BM cells), which can lead to both off-target effects and misinterpretation of data. The review by Chen et al. (2017) details these limitations with respect to understanding the roles of various components of the BM niche in adult settings, and emphasizes the need for validation of these tools.^101^ Meanwhile, HSC-specific fluorescent reporter-based tracing (e.g., α-Catulin-GFP as a marker) combined with deep optical clearing shows that ~85% of HSCs reside within 10 μm of sinusoids and are largely excluded from arterioles, challenging earlier arteriolar-centric models.^17^

Recent experimental systems are beginning to disentangle local versus systemic determinants of HSC regulation. In a recent study, Takeishi et al. engineered a femur-transplantation system to increase the in vivo available HSC niches.^102^ They found that, despite increasing the number of niches, the total HSC numbers did not expand, suggesting the presence of a systemic upper limit on HSC abundance. By combining parabiosis and non-conditioned HSC transfer after bone transplantation, the authors confirmed that HSC number is constrained at both systemic and local levels. Mechanistically, titration of thrombopoietin (TPO) in a variety of Tpo^+/+^, Tpo^+/−^, Tpo^-/-^, and Tpo-transgenic hosts established circulating TPO as a systemic determinant of HSC abundance, even with expanded local niches. This work exemplifies how engineered and transplantation-based models can be used as powerful tools to functionally uncouple niche availability from systemic regulation.

Looking forward, engineered niche models offer a promising strategy to resolve some of these questions. Recent engineered marrow models incorporating vascular and stromal components have been able to support long-term HSC function ex vivo in the absence of exogenous cytokines,^103^ and micro-engineered vascular niche organoids have also been developed to more precisely functionally interrogate endothelial vs stromal contributions to HSC fate decision.^104^ These engineered systems promise to shift the field from correlative aging signatures to causal hierarchies and help understand whether vascular rarefaction, neuronal loss, inflammatory dysregulation, or some other factors act as initiating events in niche aging.^45^

Ultimately, the aged BM niche is altered in many dimensions, including the vasculature, stroma, immune compartment, innervation and whole body physiology.^105^ From a translational standpoint, this implies that there are at least two complementary intervention strategies: targeting the surrounding microenvironment (e.g., reversing vascular decline or decreasing inflammation) or directly addressing HSC-intrinsic defects (e.g., stem-cell replacement, genetic reprogramming). To disentangle these complex overlapping mechanisms, temporally and systemically resolved experimental tools will be essential, including in vivo models of aging, defined engineered niches, and manipulations at different levels of organ and cellular compartmentalization, in a logical sequence that will allow us to establish cause and effect.

## Leukemia and the BMN: driver, accomplice, or passive bystander

**Fig. 4:**
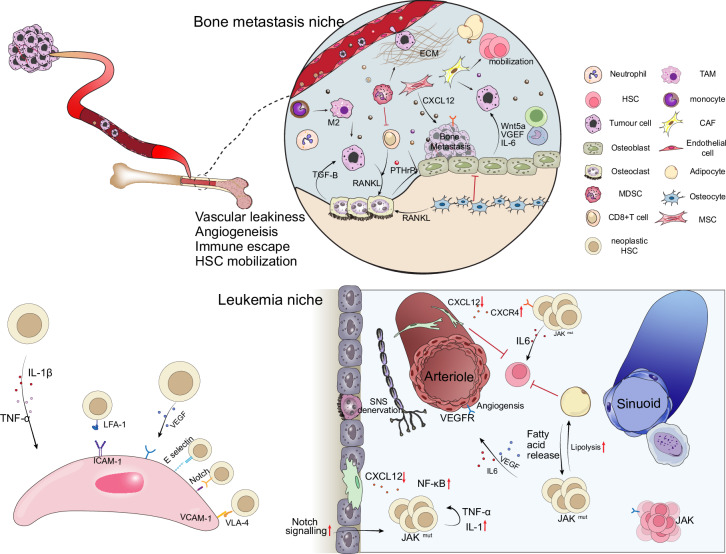
Malignant bone marrow niche during bone metastasis and leukemia. During bone metastasis, primary tumor cells and tumor-derived microRNAs migrate through the circulation while elevating systemic inflammation. Upon reaching the bone marrow niche, they exploit vascular leakiness and angiogenesis to infiltrate and establish a metastatic niche. Tumor–niche interactions promote immune suppression and HSC mobilization via Osteoclast activation via RANKL signaling, which leads to bone resorption. The extracellular matrix and osteolineage surfaces serve as anchoring sites that support tumor colonization. In leukemia, neoplastic HSCs with JAK mutations remodel the niche by releasing IL-6 and VEGF-driven angiogenesis. They lead to adipocytes' lipolysis and activation of Notch signaling, maintaining leukemic self-renewal. Concurrently, CXCL12 expression is reduced, disrupting normal HSC maintenance and favoring malignant niche dominance

As with aging, a central unresolved question in hematologic malignancies is the nature of the relationship between the leukemic clone and the BMN: is the niche a passive responder to leukemic infiltration, or does it play an active role in initiating or supporting malignancy? This “chicken-or-egg” dilemma remains one of the most vigorously debated topics at the interface of skeletal biology and hematopoietic malignancy. Fig. 4 contrasts *niche-initiated* malignant evolution with *tumor-driven* remodeling.

Evidence from elegant murine models has demonstrated that primary dysfunction in bone marrow stromal cells can initiate malignant hematopoiesis, even in the absence of hematopoietic cell-autonomous mutation.^25,106^ For instance, deletion of Dicer1 specifically in osteoprogenitor cells induced a myelodysplastic syndrome (MDS)-like phenotype that progressed to acute myeloid leukemia (AML). Importantly, transplantation of healthy HSCs into this aberrant niche was sufficient to induce disease, providing compelling evidence for niche-driven oncogenesis.^106^ These findings support the idea that a disordered microenvironment, particularly one originating from skeletal-lineage cells, can promote clonal evolution and malignant transformation by inducing genotoxic stress or altering HSC selection dynamics.

Conversely, as described above for inflammaging and vascular rarefaction, there is extensive literature indicating that leukemic cells are adept at remodeling the BMN and “hijack” the BMN to establish a self-reinforcing environment that favors their own survival and proliferation.^107–110^ In AML, for example, leukemic blasts secrete inflammatory cytokines, chemokines, and extracellular vesicles (including exosomes) that reshape the stromal landscape.^111^ One notable study demonstrated that AML-derived exosomes impaired osteolineage differentiation and promoted the expansion of undifferentiated stromal progenitors through upregulation of DKK1, a Wnt pathway antagonist, and suppression of CXCL12, SCF, and IGF-1, which are essential for HSC maintenance.^112^ Fig. 4 highlights the feed-forward loop by which leukemic cues suppress HSC-support factors such as CXCL12 and stabilize a leukemia-permissive niche.

However, the temporal sequence of these events remains debated, echoing the challenges also noted in the process of aging. Do leukemic cells remodel the niche early, even during pre-leukemic stages, or do these changes emerge only after malignant infiltration? Some preclinical models suggest that niche remodeling precedes overt disease.^113^ For example, in MLL-AF9-driven leukemia, early depletion of osteoblasts and increased vascular permeability were observed before full leukemic transformation.^114,115^ These findings raise the possibility that pre-leukemic clones may actively condition the niche to create a permissive environment for transformation and expansion. Other reports argue that remodeling is secondary to high leukemic burden. For example, AML patients exhibit progressive functional impairment in niche stromal cells, such as osteolineage suppression and stromal activation, that correlate with disease burden and predict outcome.^111^ Systemic factors can also influence this interaction; for instance, chronic inflammation, often associated with aging, can predispose the niche to malignant transformation by reprogramming stromal cells toward pro-inflammatory phenotypes and impairing their osteogenic potential through IL-1β, TNF-α, and IFN-γ signaling.^78^ Moreover, elevated oxidative stress, as seen in aging and chronic inflammation, can lead to DNA damage and activate stress pathways leading to exhaustion of HSCs,^116^ while clones (or leukemic stem cells) enriched in lower ROS states can gain a selective advantage in this environment^117–119^ that facilitates their persistence and expansion.

This dynamic and bidirectional interaction between leukemia and the niche has generated significant interest in targeting niche-leukemia crosstalk as a therapeutic strategy^120^ besides strategies that directly aim to ablate AML blasts or LSCs by exploiting intrinsic vulnerabilities.^121–129^ The CXCL12–CXCR4 axis, critical for retaining both HSCs and LSCs in protective niches, has been a major focus.^130,131^ Inhibition of CXCR4 using antagonists like plerixafor has been shown to mobilize LSCs out of their protective microenvironment and sensitize them to chemotherapy in preclinical models.^132,133^ Similarly, disrupting other adhesive interactions (e.g., VLA-4/VCAM-1) or blocking growth factor signaling pathways (e.g., SCF–c-Kit) has shown therapeutic potential.^133–135^ Parallelly, niche-restoring strategies have also been explored. For example, inhibition of inflammatory mediators such as TNF-α or restoration of TGF-β signaling has been reported to partially reverse leukemic niche remodeling.^136^ Targeting niche-derived factors such as DKK1 not only promoted osteolineage recovery but also delayed leukemia progression in murine models, suggesting that normalizing the stromal environment can constrain disease.^112^

Despite these advances, the field remains divided over the therapeutic relevance and feasibility of niche-centric interventions.^137^ Some argue that niche remodeling is largely a secondary phenomenon driven by high leukemic burden and will naturally resolve with effective leukemia clearance [NCT03616470]. From this view, direct targeting of the niche is unnecessary. Others contend that the niche becomes an active co-conspirator in disease progression and relapse, forming a pathological feedback loop that must be interrupted for a durable therapeutic response.^108,110,135^ This perspective is supported by the observation that niche alterations, particularly inflammatory and osteogenic suppression, can persist even after cytoreduction of leukemic cells,^113,137^ reinforcing the need for therapies that address both leukemic and stromal compartments.

As niche-directed therapies move into clinical testing, their outcomes will be critical in determining whether the BMN is a modular, druggable component of the leukemic ecosystem or merely a passive casualty of malignant expansion. Resolving this controversy has profound implications for skeletal biology, regenerative medicine, and hematologic oncology alike.

## The BMN as a systemic sensor and effector

The BMN is not isolated but is deeply interconnected with systemic physiology. Hormonal cues, inflammatory signals, neural innervation, and metabolic status all shape the behavior of bone marrow stromal and endothelial cells, thereby influencing HSC fate.^1,2,138,139^ Increasing evidence reveals that systemic factors exert powerful, and often reversible, effects on the BMN, particularly its bone-forming components such as osteoblasts and osteoprogenitors.^140^

Disruption of this adrenergic innervation not only alters HSC mobilization but has also been implicated in premature HSC aging.^86^ Similarly, systemic administration of G-CSF, commonly elevated during infection, suppresses CXCL12 via niche macrophage signaling, thereby disrupting osteoblast-dependent HSC retention and accelerating myeloid output.^141,142^ These findings establish the concept that systemic stressors such as infection and circadian stress act through bone-resident cells to reshape the hematopoietic output.^54^

Building on the inflammation described earlier, chronic systemic inflammation plays a key role in niche dysfunction. The increased circulating IL-1β, IL-6, TNF-α, and type I interferons signal to stromal and endothelial cells to disrupt HSC quiescence and skew hematopoiesis toward myeloid-biased and dysfunctional outputs.^77,78,142^ Acute inflammatory triggers, including experimental LPS exposure, induce vascular remodeling marked by increased permeability and reduced CXCL12 expression, further illustrating how systemic inflammation rapidly reshapes the niche.^142–144^ In the long term, this inflammatory remodeling contributes to HSC exhaustion and facilitates the emergence of clonal hematopoiesis.^145–147^

Recruitment of circulating myeloid cells back to the bone marrow is another layer of systemic local integration. After migrating to peripheral tissues to perform effector functions, senescent neutrophils upregulate CXCR4 and home back to the marrow in a CXCR4–CXCL12–dependent manner, where they are cleared by marrow macrophages.^147–149^ This process of neutrophil reuptake follows a circadian pattern that is coordinated by adrenergic regulation of CXCL12, thereby coupling neutrophil clearance to HSC mobilization and niche remodeling.^150^ Classical monocytes can also home to the marrow in response to CXCR4 gradients, and either die in the marrow or differentiate into marrow macrophages, and represent another potential link between peripheral inflammatory activity and central stromal remodeling.^150–152^ Through these reciprocal pathways, the BMN serves as a sensor and quality-control center, in addition to a site of immune cell production, that integrates systemic immune experience to regulate hematopoietic output. Dysregulation of this circuit results in impaired immune clearance, persistent inflammatory input, and maladaptive stress on hematopoiesis.^153^

Systemic metabolism and diet also profoundly influence the bone marrow milieu,^154–156^ while caloric restriction or exercise enhances bone mass and improves HSC function, in part via sympathetic tone and reductions in ROS.^157,158^ Short-chain fatty acids (SCFAs) derived from the gut microbiota can influence hematopoiesis and niche cytokine expression; direct CXCL12/SCF modulation has been suggested but remains under investigation.^159,160^

Age-associated hormonal changes are another potential widespread driver of niche dysfunction. The aging-associated decline in sex steroids (estrogen in women with menopause and testosterone in men with andropause) is a major contributor to age-related bone loss and has also been shown to perturb bone marrow homeostasis.^161^ The loss of sex hormones enhances inflammatory cytokine production (IL-6, IL-1β, and TNF-α), which can directly affect myelopoiesis, as well as stromal adipogenesis and reduced^162–164^ osteoblast function to skew the hematopoietic program towards myeloid bias and bone loss. In addition to decreasing sex steroids, pituitary-derived FSH has also been identified as a systemic pro-aging signal acting on both skeleton and adipose tissue; monoclonal antibodies targeting FSHβ subunits increase bone mass (with accompanying anti-adiposity effects demonstrated in related studies).^165^ Administration of a humanized FSH-neutralizing antibody has, in fact, been shown to target bone and fat in vivo, arguing for the druggability of this pathway.^166^ Parathyroid hormone (PTH), which acts as an osteoblast differentiation factor and can enhance niche-derived support for HSCs, can also stimulate hematopoietic recovery when delivered intermittently in preclinical models.^167^ Osteolineage cells have thus emerged as a hormone-sensitive niche component regulating hematopoietic quiescence. Growth hormone and IGF-1 deficiency with age (“somatopause”) also impairs osteoblast function and HSC support.^168^ Hormonal dysregulation of glucocorticoids, which exhibit diurnal and age-associated shifts in their expression, also drives aging-related changes to the composition and function of the niche. Glucocorticoids contribute to age-related remodeling of the bone marrow niche in part by suppressing osteoblast differentiation and bone-forming capacity.^169,170^ Beyond the traditional endocrine axes, systemic metabolic and lysosomal conditions can also program the marrow–bone unit. For example, mutations in glucocerebrosidase (GBA1) causing Gaucher disease alter lipid handling and macrophage/osteoclast biology to dysregulate bone and marrow homeostasis.^171^ Oxytocin and other pituitary/neuroendocrine hormones have been demonstrated to exert coordinated control over bone remodeling and adipose biology as well.^172^ Hormone replacement strategies with PTH analogs (teriparatide), estrogen/androgen supplementation, and/or thyroid hormone optimization are clinically validated for the treatment of bone and metabolic disease and are an easily accessible avenue for niche rejuvenation, although their specific impact on hematopoietic reconstitution has not yet been defined.^167,173,174^ Overall, these data support the endocrine system as an important driver of niche aging that may be susceptible to manipulation to restore bone marrow function.

Environmental toxins are now emerging as novel systemic modulators of bone marrow health.^175^ A recent study demonstrated that orally ingested microplastics disrupt gut barrier integrity, leading to systemic inflammation and niche dysfunction, ultimately impairing HSC self-renewal.^175^ These findings suggest that environmental exposures can indirectly impact skeletal and hematopoietic health through gut-mediated systemic pathways.

Despite these insights, the question remains unresolved as to whether systemic or local niche cues primarily govern HSC behavior. For example, transplantation experiments reveal that even young, functional HSCs adopt aged phenotypes when placed in aged or fibrotic BMNs, suggesting that local cues such as osteoblastic decline, increased matrix stiffness, and pro-inflammatory stromal signaling override systemic input.^176^ This raises a fundamental question: is HSC behavior primarily governed by the bone niche microenvironment (“nurture”), or by circulating factors and systemic stress (“nature”)?

The answer likely lies in their synergy.^105^ Systemic factors ranging from neuroendocrine cues and inflammatory mediators to metabolic hormones reprogram niche cells, altering their secretome and structural properties.^105^ In turn, localized niche interactions such as SCF–c-Kit signaling, Notch–Jagged engagement, and osteoblast-derived factors act in spatially restricted manners to enforce HSC fate decisions.^16,177,178^ This duality poses a therapeutic dilemma: should interventions aim to modulate systemic inputs (e.g., anti-inflammatory strategies, metabolic correction) or directly target bone-resident niche cells (e.g., PTH analogs to stimulate osteoblasts or agents that restore endothelial CXCL12 expression)^16,54^ or maybe both?

Clinical trials have already explored both routes. G-CSF mobilizes HSCs by acting on the niche via systemic cytokine pathways,^141^ while anabolic PTH analogs have shown promise in stimulating osteoblasts to improve hematopoietic recovery.^179,180^ However, the long-term impact of such strategies remains uncertain, particularly in aged or fibrotic niches where niche plasticity may be diminished.^105^

In conclusion, the BMN operates at the interface of systemic and local regulation. Understanding how these layers interact will be essential to designing therapies that rejuvenate the aged niche, restore hematopoietic balance, and improve outcomes in marrow failure, osteoporosis, and hematologic malignancies.

## Skull bone marrow in aging: a site of degeneration or resilience

The calvarial skeleton is perceived as a resilient hematopoietic niche; however, mounting evidence challenges this view.^181^ However, the skull undergoes pronounced age-associated deterioration, and both calvarial bone and marrow are profoundly altered in age-related neurological conditions.^181–184^ Aging is accompanied by bone loss, cortical thinning, and structural remodeling, indicating that the cranial skeleton is not spared from degenerative processes.^181–183,185^ Within this context, the notion that calvarial bone marrow constitutes a uniquely expanding or resilient hematopoietic reservoir during aging is not supported by the bulk of available evidence. Across diverse experimental platforms—including imaging, proteomics, and single-cell transcriptomics—a consistent pattern emerges: the calvarial marrow niche undergoes multifaceted degeneration with age. As mesenchymal stem and osteoprogenitor populations decline, angiogenic programs are suppressed. Canonical hallmarks of aging, including DNA damage, replication stress, mitochondrial dysfunction, cellular senescence, and chronic inflammation, become increasingly prominent in the aging skull.^181–186^

Claims of skull marrow resilience during aging by Adams et al.^187^ have largely relied on technical approaches that may incompletely capture marrow architecture. Imaging of uncleared whole-mount calvaria using confocal microscopy is inherently limited by restricted penetration depth, leading to incomplete visualization of marrow cavities and regional heterogeneity. Similarly, analyses based on whole-skull preparations across imaging, single-cell sequencing, and ELISA assays risk conflating marrow-derived signals with contributions from cortical bone and surrounding tissues. Apparent preservation or expansion of vascular or hematopoietic features may therefore reflect methodological artefacts rather than true niche resilience and bone marrow preservation. Even in the Adams et al. study that claims calvarial marrow is resilient, a re-examination of their single-cell RNA-sequencing data contradicts this conclusion.^187^ Their published UMAP plots show a clear reduction in endothelial cells in aged calvarial samples compared with young samples.^187^ Therefore, the authors’ own data demonstrate age-related loss of endothelial cells, not preservation or expansion as they claim.

Viewed within a broader skeletal framework, the calvarial bone marrow and vascular niches do not appear exceptional or uniquely preserved during aging. Aging trajectories diverge across anatomical sites: vertebral marrow—the principal hematopoietic reservoir in adults, exhibits relative preservation of key hallmarks of aging, including excessive DNA damage, senescence, and inflammatory signaling.^181^ In contrast, both calvarial and long-bone marrows show accelerated degeneration. This skeletal site-specific vulnerability highlights that bone marrow aging is not uniform. The consequences of calvarial marrow degeneration may extend beyond hematopoiesis.

Given its direct vascular connections to the meninges, age-associated deterioration of the skull niche could disrupt neuroimmune surveillance and exacerbate neuroinflammatory processes.^183^ Such changes may influence the progression of neurodegenerative diseases and age-related cognitive decline, placing the aging calvarial marrow at the intersection of skeletal, immune, and neural aging. Clinically, the relative resilience of vertebral marrow may explain why many older individuals retain sufficient hematopoietic reserve for transplantation. Collectively, these findings argue against the notion of a uniquely resilient skull marrow. The accelerated deterioration of calvarial marrow relative to vertebrae exemplifies this heterogeneity and identifies senescence, replication stress, and mitochondrial dysfunction as actionable drivers of niche failure.

Additional findings reveal classical hallmarks of niche aging in the skull: a decline in lymphoid progenitor output, skewing toward myeloid-biased hematopoiesis, deterioration of stromal components, including fibrotic remodeling,^182^ and age-related loss of peripheral innervation. These observations are consistent with niche dysfunction patterns seen in long bones and, in some cases, may be more pronounced in the calvarium. Interestingly, although neurovascular rarefaction is seen in the aged skull, the spatial association between peripheral nerves and Type-H vessels in particular is maintained. Recent work further indicates that aging skull marrow frequently degenerates, with progenitor attrition, suppressed angiogenic/lymphatic signatures, and barrier dysfunction that amplify neuroimmune crosstalk via skull–meningeal conduits, tempering the view of the calvarium as uniformly resilient.^181^ Taken together, these observations reflect an open question in the field: do certain marrow sites exhibit intrinsic resistance to age-related degeneration, or do all niches ultimately succumb to systemic and local aging cues? The calvarial marrow thus represents an important model for further investigation into site-specific resilience versus vulnerability in the aging bone niche landscape.

## Rejuvenating the aged niche: translational strategies in hematology, regeneration, and cancer

As discussed throughout this review, defining the drivers of BMN aging is not just an academic question; it has direct clinical implications. Age-related alterations in the BMN contribute to anemia, immune dysfunction, delayed hematopoietic recovery, and increased incidence of hematological malignancies.^96,188–192^ These changes impair the capacity of the niche to maintain hematopoietic stem-cell (HSC) function and disrupt its regulatory role in maintaining homeostasis. Based on the aging-associated mechanisms discussed above, Table 2 provides a structured overview of key signaling pathways disrupted in the aging bone marrow niche, their primary cellular targets, and corresponding therapeutic entry points that are being explored to restore niche function.

**Table 2:** Aging-associated pathways and therapeutic strategies in the bone marrow niche

Aging pathway/process	Molecular targets	Therapeutic approach	Development stage	Reference
Chronic inflammaging	IL-1R, TNF-R, IFN-R, NF-κB, NLRP3	IL-1 receptor antagonists (anakinra), TNF inhibitors (etanercept), anti-IFN antibodies	Preclinical + Clinical trials for other indications	^78,145^
Cellular senescence	BCL-2 family, p16/p21, SASP factors	Senolytics: dasatinib + quercetin, navitoclax, fisetin	Preclinical + early Phase I/II	^194,197^
Vascular dysfunction	VEGF, Notch, Ang-Tie2, CXCL12, SCF	Endothelial transplantation, VEGF modulation, Notch agonists, young EC transfer	Preclinical	^73,95^
Sympathetic denervation	β2/β3-adrenergic receptors	β3-AR agonists (CL-316243, mirabegron)	Preclinical; mirabegron FDA-approved (overactive bladder)	^86^
ECM stiffening & fibrosis	Collagen crosslinks, TGF-β, LOX, matrix stiffness	Compliant hydrogel niches, LOX inhibitors, TGF-β inhibition, BAPN	Preclinical	^49,214^
HSC retention/mobilization	CXCR4, VLA-4, c-Kit	CXCR4 antagonists (plerixafor/AMD3100), G-CSF	FDA-approved (HSC mobilization)	^141^
Osteoblast decline	PTH receptor, Wnt/β-catenin	PTH analogs (teriparatide, abaloparatide), Wnt agonists	FDA-approved (osteoporosis); tested for HSC engraftment	^179,180^
Mitochondrial dysfunction & ROS	ROS, mitochondrial integrity	Mitochondria-targeted antioxidants (MitoQ, SS-31), NAD^+^ precursors (NMN, NR)	Preclinical	^205^
DNA damage	p53, DNA repair pathways	PARP modulators, DNA repair enhancers, antioxidants	Preclinical, Clinical	^230,231^
Adipose accumulation	PPARγ, adipokine signaling	PPARγ inhibitors, anti-adipogenic therapies, exercise/metabolic modulation	Preclinical	^232,233^
Autophagy decline	mTOR, ULK1, autophagy regulators	mTOR inhibitors (rapamycin), caloric restriction mimetics	Preclinical; rapamycin in clinical aging trials	^202,234–236^
Epigenetic drift in HSCs and niche	DNA methyltransferases, HDACs, TET enzymes	HDAC inhibitors, DNA methylation modulators, vitamin C (TET activation)	Preclinical/clinical (cancer context)	^237,238^
Metabolic reprogramming/nutrient sensing	mTOR, AMPK, insulin/IGF pathways	Rapamycin, metformin, caloric restriction mimetics	Preclinical and clinical aging trials	^236,239,240^

Development stages are approximate and may vary by specific compound. Many preclinical strategies show promise but require validation in human aging contexts. Combinatorial approaches targeting multiple pathways simultaneously may be necessary for meaningful BMN rejuvenation

Several promising therapeutic strategies are emerging to rejuvenate or reprogram the aged niche, which can be categorized by their mechanism of action. Senolytic drugs such as dasatinib+quercetin, fisetin, and navitoclax (ABT-263) clear senescent MSCs and endothelial cells through inhibition of anti-apoptotic BCL-2 family proteins and ablation of the cellular source of SASP (IL-6, IL-1β, TNF-α) factors that mediate inflammaging.^193–197^ In aged mice, senolytic treatment re-established expression of CXCL12 and SCF by stromal cells, ameliorated vascular leakiness, and improved HSC repopulation capacity, and senolytics are now in clinical trials for age-related diseases (NCT02848131, NCT04313634).^198,199^ Targeted anti-inflammatory therapeutics, such as IL-1 receptor antagonists (anakinra, canakinumab), block emergency myelopoiesis and restore HSC quiescence,^78,200^ and TNF-α inhibition is likely to be beneficial in settings of chronic, pathological elevation, although context and timing are critical given TNF-α‘s context-dependent effects on immune surveillance.^201,202^ Pro-angiogenic and osteogenic agents act on a structural niche deficit. PTH analogs (teriparatide, abaloparatide) promote osteoblast function through activation of PTH1R and have the capacity to expand HSC niches and improve engraftment after transplantation.^179,180,203^ Anti-sclerostin antibodies (romosozumab) inhibit sclerostin to promote osteoblast activation and bone formation by activating Wnt signaling.^204^ The restoration of Type-H vasculature through local activation of Notch or VEGF signaling is in preclinical development and may be incorporated into tissue engineering approaches,^43,45^ although systemic pro-angiogenic strategies are likely to be unsafe. Lymphangiogenic strategies to repopulate aged bone marrow with lymphatic vasculature include transplantation of young lymphatic endothelial cells or administration of VEGF-C.^61,71^

Metabolic and neuroendocrine optimization approaches may target upstream systemic drivers of niche aging. Targeted antioxidants delivered to mitochondria (MitoQ, SS-31/elamipretide) reduce ROS levels in HSCs and stromal cells to improve their function in aged models.^205–207^ Caloric restriction mimetics, such as mTOR inhibitors (rapamycin, everolimus), metformin, and NAD^+^ precursors (nicotinamide riboside, NMN), act on pathways of autophagy and stress resistance to protect against cellular aging.^208–210^ The randomized placebo-controlled TAME (Targeting Aging with Metformin) trial will be the first clinical trial with geroprotective agents, including hematopoietic parameters, to provide clinical readouts of such agents.^211^ Restoration of sympathetic tone with the β3-adrenergic agonist mirabegron has been shown to improve niche integrity and HSC function in denervated or aged mice,^86^ and could be a repurposing opportunity for this FDA-approved drug for overactive bladder. Cell replacement strategies, including transplantation of young MSCs, endothelial cells, or lymphatic endothelial cells into aged hosts, directly replenish niche components and have proven efficacy in preclinical models, with challenges remaining regarding cell source, delivery, engraftment, and long-term persistence.^160,203^

Importantly, the senescent niche phenotype involves a network of related deficits in inflammation, vasculature, metabolism, and innervation, and suggests that strategies combinatorially targeting several of these mechanisms at once or in sequence would be of benefit. Examples of rational combinatorial strategies might include the pairing of senolytics with pro-angiogenic factors to simultaneously eliminate malfunctioning cells and repair vascular structures, or the pairing of anti-inflammatory molecules with PTH or analogs to both reduce inflammatory tone and promote osteogenesis.^212^ However, as in other settings, such interventions will need to be carefully titrated and timed in the context of the mixed roles of each factor: chronic IL-1 signaling contributes to HSC exhaustion, but acute IL-1 stimulation supports regeneration in response to injury;^78^ TNF-α limits self-renewal, but also has a role in immune surveillance;^201^ and TGF-β supports quiescence, but high levels lead to fibrosis.^136^ This would benefit from a precision medicine approach that profiles patients into distinct “niche endotypes” that are inflammatory-dominant, vascular-depleted, metabolically disrupted, or other, based on circulating markers of inflammatory state, angiocrine factors (CXCL12, SCF), vascular leakage, or imaging-based metrics of marrow adiposity or vascular density.^51,64,65^ Validated biomarkers to support this, combined with patient-specific ex vivo models of the niche, such as iPSC-derived organoids for personalized drug screening,^213–215^ would be essential to move preclinical concepts of niche-directed therapies into clinical practice. Bridging these basic biology insights into a patient-specific framework will be key to translating bone marrow niche rejuvenation from a therapeutic goal into a clinical reality.

## Broader context and challenges

Our current understanding of the BMN (BMN) has been shaped largely through studies in murine models. Mice offer unparalleled experimental tractability, particularly for in vivo lineage tracing, genetic perturbations, and high-resolution imaging. These approaches have illuminated the structural and functional complexity of BMN.^1^ However, the translational relevance is constrained by species-specific differences. Biologically, murine marrow exhibits a dominant sinusoidal vasculature. In contrast, human marrow is more trabecular, interspersed with fibrotic and adipocytic zones, and marked by greater interstitial heterogeneity.^6,14,44^ Furthermore, well-characterized murine niche populations such as CXCL12-abundant reticular (CAR) cells, Nestin^+^ MSCs, and leptin receptor-positive (LepR^+^) stromal cells remain incompletely defined in human tissues. This is due to limited sample access and the difficulty of preserving spatial integrity during tissue processing.^204^ Indeed, multiple interventions, including G-CSF–induced mobilization or niche-targeting antibodies, have shown promise in mouse models but have failed to deliver comparable clinical efficacy.^78^ This reflects a gap in physiological relevance and human-specific validation.

Technically, intravital imaging of bone is hampered by photon scattering, bone autofluorescence, contribution from cortical vessels, and spatial inaccessibility, especially in deeper marrow regions. Even pioneering work using two-photon live imaging of calvarial marrow demonstrated that only superficial regions can be reliably visualized, highlighting both the power and the limitations of this approach.^216,217^ Although single-cell and spatial transcriptomic technologies have expanded our cellular census of the BMN, they have also exposed technical and conceptual challenges. Stromal cells, particularly MSCs and endothelial subtypes, yield low RNA quantities, are sensitive to enzymatic dissociation, and are susceptible to ex vivo artifacts. As a result, datasets can vary significantly between platforms and laboratories, impeding cross-comparative analyses. In humans, where lineage tracing is not feasible, these limitations are further compounded, leaving substantial gaps in our functional understanding of niche hierarchies and cell–cell interactions.

In addition to technical barriers, the field faces conceptual divergence across disciplines. Hematologists, immunologists, and stem-cell biologists approach the BMN through different lenses, shaped by distinct priorities, tools, and clinical endpoints. Investigators emphasize different aspects of the niche, from stromal cytokine support to immune-inflammatory cues to mechanical and neural regulation. These divergent frameworks shape not only experimental design but also terminology, for example, Nestin^+^ MSCs (stem-cell biology) versus LepR^+^ MSCs (hematology) may describe overlapping populations yet are conceptualized in distinct frameworks.^11^ There are several overlapping terms used to define perisinusoidal stromal cells. These include CXCL12-abundant reticular (CAR) cells (operationally defined based on CXCL12 expression), LepR^+^ cells (operationally defined based on leptin receptor expression), and Nestin^+^ MSCs (operationally defined based on expression of a nestin-GFP reporter). These populations are highly overlapping but not completely equivalent. LepR^+^ cells are the most inclusive population, whereas CAR cells define cells based on a functional readout. Variable expression depending on context and experimental variation has led to some controversy over hierarchy and cell identity. In this review, we have used the term LepR^+^/CAR/Nestin^+^ to identify perivascular MSC-enriched fractions, even though different markers are employed in the hematology, stem-cell biology, and skeletal biology literature. This disciplinary fragmentation has practical implications. Cytokine perturbations such as IL-1 or TNF-α exposure may be interpreted as emergency hematopoiesis, HSC exhaustion, or myeloid skewing depending on the investigator’s lens. Such heterogeneity in interpretation hampers the formation of unified mechanistic models and clouds translational strategies.

## Engineered bone marrow niche models: spatial organization and co-culture systems

A major limitation of BMN studies is the lack of ability to precisely manipulate and spatially pattern the major niche cell populations, bone marrow endothelial cells (BMECs), lymphatic endothelial cells (LECs), and mesenchymal stromal cells (MSCs), in a controlled manner. Tissue-engineered BMN models are bridging this gap through increasingly sophisticated co-culture and spatial patterning approaches.^80,81^ Bone marrow organoids derived from induced pluripotent stem cells (iPSCs), incorporating endothelial and mesenchymal components, represent a promising advance for modeling hematopoietic and leukemic niches.^213^ Engineered hydrogels that mimic fibronectin-rich ECM environments (e.g., PeptiGel) have supported functional studies of HSC maintenance and leukemic stem-cell resistance.^214^ Vascularized organoid and organ-on-chip systems provide additional control over environmental variables.^215^ Recently, Crown Bioscience’s 3D BMN platform, presented at AACR 2025, demonstrated robust ex vivo modeling of acute myeloid leukemia and multiple myeloma, with integrated high-content imaging for drug response profiling [Crown Bioscience, AACR 2025]. Beyond hematological malignancies, the metabolic microenvironment chamber (MEMIC), a 3D-printed ex vivo platform developed to model intratumoral heterogeneity in solid tumors, has provided proof-of-concept for recapitulating the metabolic and vascular complexity of malignant niches.^215,218^ The MEMIC has not been implemented in AML to date, but the system could be modified in the future to help study the impact of disrupted vasculature and metabolic gradients on LSCs.

These spatially organized co-culture platforms are finding use as tools to model BMN aging and/or screen potential regenerative approaches. For example, the inclusion of senescent MSCs in the perivascular space reproduces several features of in vivo aging: decreased CXCL12 secretion, SASP factor expression that impairs neighboring BMECs, and reduced support of HSCs.^20^ Aged BMECs with age-related deficits in angiocrine factor expression and increased vascular permeability can also be co-cultured with young or aged MSCs to further parse out cell-autonomous aging from niche-mediated changes.^41^ Inclusion of LECs, whose function in the aging BMN is not well understood^61^ will allow investigation of lymphatic contribution to inflammaging, altered immune cell trafficking, and potential lymphatic dysfunction in tissue regeneration. For the latter purpose, these systems also enable a more physiologically relevant test bed for cellular therapies (transplantation of young BMECs, rejuvenation of MSCs), pharmacological treatments (senolytics, anti-inflammatory drugs), or matrix modifications (softening, ECM remodeling).^40,155,156,164^ Missing from these spatially organized co-culture platforms are immune cell dynamics, neural innervation, and circadian rhythm, as well as maturation issues in long-term cultures. In general, spatially organized co-culture systems offer an important experimental middle ground, where the cell–cell, niche architecture, and aging mechanisms difficult to interrogate in vivo can be dissected mechanistically and in a scalable fashion for therapeutic screens.^80,81,163–165^

While these humanized systems are still in the early stages, they offer an opportunity to develop physiologically faithful models for mechanistic discovery, therapeutic screening, and personalized medicine. Ultimately, addressing these broader challenges, species-specific differences, technological barriers, and disciplinary silos will be essential for developing a unified, clinically actionable understanding of the BMN.

## Conclusion

The BMN has evolved from a static scaffold to a recognized master regulator; a dynamic, hierarchical microenvironment whose disruption during aging drives cascading failures across hematopoiesis, immunity, and skeletal homeostasis. Recent technical innovations have uncovered unimaginable complexities in niche organizations, as well as revealed important knowledge gaps. Together, the available data are consistent with a model whereby vascular integrity, stromal lineage bias, neural regulation, and chronic inflammatory tone are an interconnected set of the core drivers of BMN aging. All of these create positive feedback loops, vascular dysfunction results in inflammation, which causes endothelial damage and stromal senescence; SASP is increased by senescent cells; denervation weakens the vasculature; and a stiffened matrix limits tissue remodeling. Identifying initiating events remains critical, as early interventions at key bottleneck steps could prevent an entire downstream cascade.

Several unresolved questions demand integrated experimental approaches. An outstanding question that needs to be addressed is the temporal hierarchy of events that lead to vascular rarefaction vs. inflammaging. While there are a few longitudinal datasets that provide hints that loss of Type-H vessels can be an early event in aging,^45^ large-scale time-resolved imaging studies^15,41,42,162^ will be required to resolve the temporal order of events. A second important question is whether the events are more cell-intrinsic or niche-driven. Aged HSCs transplanted into young niches display transcriptomic rejuvenation but also maintain several functional defects, including myeloid bias and defects in self-renewal,^76,77^ pointing to a dual mechanism whose therapeutic targets are not clear to reverse.^82,150^ All these mechanisms have been studied mainly in mice and therefore need validation in humans, particularly because of potential species differences in niche anatomy and pathologies. Finally, there is emerging evidence for possible anatomical heterogeneity and resilience that can be site-specific, for example, in calvaria vs. long bones vs. vertebrae.^144,145,147^

Looking forward, an important consideration for developing rejuvenation therapies is that these are likely to be combination therapies and need to be personalized. As aging is a multifactorial process, it is likely that the combination of senolytics^175^ with anti-inflammatory^57^ and pro-angiogenic therapies will be needed to have a synergistic rejuvenative effect. Furthermore, development of non-invasive biomarkers (circulating angiocrine factors, circulating inflammatory signatures, or imaging-based vascular metrics)^47,60,61^ will be critical for monitoring the effects of these therapeutics in a clinical setting, and also for stratifying patients into endotypes of aging, to best select therapeutic regimens for each patient: e.g. some patients may be more inflammatory-prone and respond better to an IL-1 blockade, while other patients may be more vascular-deficient and require more pro-angiogenic therapies that target endothelial restoration. Furthermore, ex vivo models of BM,^104^ such as engineered BMN organoids, will be required for personalized drug screening for each individual patient. To move from complexity to clarity, however, will require more than just defining niche components. It will require the identification of causal hierarchies, temporal sequences, and actionable biomarkers that stratify individual patients into personalized combination therapies. Only with the combination of mechanistic precision and clinical heterogeneity will we make rejuvenation of the bone marrow niche move from wish to reality, so that we can rebuild the foundation of healthy aging for blood and bone.
